# Optimization of lipase production using fungal isolates from oily residues

**DOI:** 10.1186/s12896-021-00724-4

**Published:** 2021-11-10

**Authors:** Leticia Miranda Cesário, Giovanna Pinto Pires, Rafael Freitas Santos Pereira, Elisabete Fantuzzi, André da Silva Xavier, Servio Tulio Alves Cassini, Jairo Pinto de Oliveira

**Affiliations:** 1 Federal University of Espírito Santo, Alto Universitário, S/N Guararema, Alegre, ES 29500-000 Brazil; 2 Federal University of Espírito Santo, Av. Fernando Ferrari 514, Vitória, ES 29075-910 Brazil; 3 Federal University of Espírito Santo, Av. Marechal Campos1468, Vitória, ES 29040-090 Brazil

**Keywords:** Fungal lipases, Optimization, Factorial design, Oily waste

## Abstract

**Abstract:**

Lipases are triacylglycerol hydrolases that catalyze hydrolysis, esterification, interesterification, and transesterification reactions. These enzymes are targets of several industrial and biotech applications, such as catalysts, detergent production, food, biofuels, wastewater treatment, and others. Microbial enzymes are preferable for large scale production due to ease of production and extraction. Several studies have reported that lipases from filamentous fungi are predominantly extracellular and highly active. However, there are many factors that interfere with enzyme production (pH, temperature, medium composition, agitation, aeration, inducer type, and concentration, etc.), making control difficult and burdening the process. This work aimed to optimize the lipase production of four fungal isolates from oily residues (*Penicillium* sp., *Aspergillus niger*, *Aspergillus* sp., and *Aspergillus* sp.). The lipase-producing fungi isolates were morphologically characterized by optical and scanning electron microscopy. The optimal lipase production time curve was previously determined, and the response variable used was the amount of total protein in the medium after cultivation by submerged fermentation. A complete factorial design 3^2^ was performed, evaluating the temperatures (28 °C, 32 °C, and 36 °C) and soybean oil inducer concentration (2%, 6%, and 10%). Each lipase-producing isolate reacted differently to the conditions tested, the *Aspergillus* sp. F18 reached maximum lipase production, compared to others, under conditions of 32 °C and 2% of oil with a yield of 11,007 (µg mL^−1^). *Penicillium* sp. F04 achieved better results at 36 °C and 6% oil, although for *Aspergillus niger* F16 was at 36 °C and 10% oil and *Aspergillus* sp. F21 at 32 °C and 2% oil. These results show that microorganisms isolated from oily residues derived from environmental sanitation can be a promising alternative for the large-scale production of lipases.

**Graphical Abstract:**

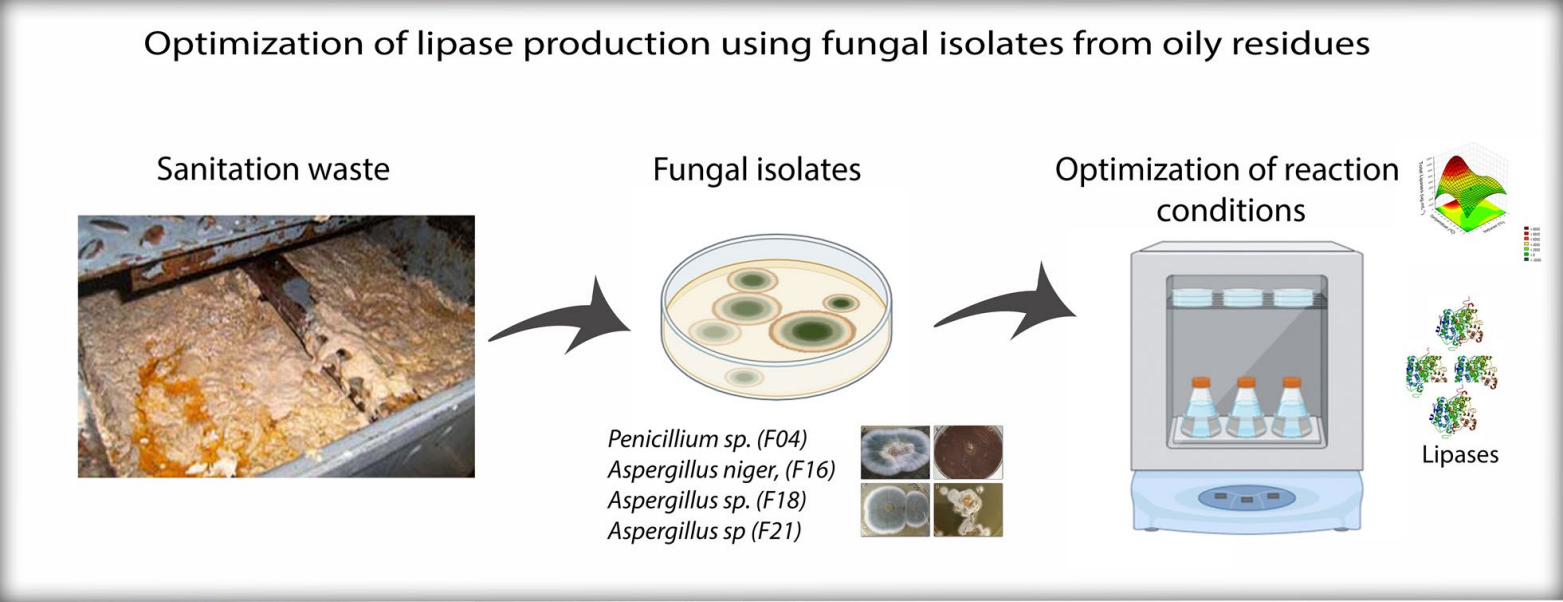

## Introduction

The enzymatic process is only one field among many more in biotechnology, although it has wide applications that are often promising alternatives when it comes to replacing conventional methodologies [1]. The employment of enzymes as biocatalysts improves the processes of production by reducing both energy and raw material costs besides generating fewer toxic residues—circumstances that are in alignment with the principles of green chemistry [2, 3]. Lipases (triacylglycerol acyl-hydrolases, EC 3.1.1.3) are enzymes with a natural role of hydrolyzing triacylglycerol (TAG) into glycerol and free fatty acids, as well as catalyzing the esterification and transesterification reactions [4, 5]. These very same enzymes play an important role in the degradation of natural materials, industrial pollutants, and other toxic products, due to two of their properties: regioselectivity and enantioselectivity [5]. For this reason, they have a potential application in the agriculture, food, detergent, leather, paper, and pharmaceutical industries [6–9].

From an economic and industrial standpoint, lipases obtained from microorganisms through the fermentation process are preferable over their animal and plant counterparts [10], given both its high yield in a relatively short amount of time and the lower costs associated with the raw materials [11]. Another economic impact is that Brazil has great potential for enzyme production because of its great biodiversity of producing organisms, even though clearly spending more on imported products [12].

Microorganisms produce extracellular lipases to hydrolyze the triglycerides in the medium, facilitating the lipid intake. The expression of microbial lipases is modulated mainly by environmental factors, such as extracellular response to a medium deprived of nutrients, variations in temperature, concentration of inductors etc. The presence of lipids and fatty acids as carbon sources induces the production of these extracellular enzymes. Microorganisms are the most interesting model for protein production, because together the repertoire of regulatory genes and constitutive promoters can be explored in the fermentation process [13, 14].

The filamentous fungi and yeasts are preferable lipase sources for commercial use because their lipases usually are part of the extracellular metabolism, which favors their extraction from the fermented medium. Another advantage is that the lipolytic fungi are considered, though not without its exceptions, to be safe microorganisms to manipulate. Apart from the increasing expectation of their application in the shape of immobilized integral lipolytic cells for reactional processes [15, 16], alternative species with potential for lipase production mostly described in the literature belong to the genera *Rhizopus* sp.*, Mucor* sp.*, Geotrichum* sp.*, Penicillium* sp. and *Aspergillus* sp. [17, 18].

Currently, the heterologous expression of coding genes is used to increase the production of lipases [19]. While this method considerably speeds-up the production of enzymes, the use of genetically modified organisms requires preparations that are not necessary for native systems. Also, in view of industrial-scale production, any surplus, such as excess nutrients and waste make the final product more expensive. With this being said, this work had as its main objective the optimization of production of lipases aiming at sustainable alternatives for the production of lipases on a large-scale A full factorial design was used to optimize the reaction conditions. The variables evaluated were temperature (°C) and inducer concentration (%), and the experiments were carried out by submerged fermentation with 4 fungal isolates from oily residues from environmental sanitation (*Penicillium* sp. F04, *Aspergillus niger* (F16), *Aspergillus* sp. F18, *Aspergillus* sp. F21).

## Metodhology

### Materials

The reagents used in the development of this work are as follows: sodium acetate (Proquimios 99%), hydrochloric acid (Sciavicco, 37%), bovine serum albumin, BSA (Sigma Aldrich, 98%), potato dextrose agar, PDA (Acumedia, 100%), calcium chloride (Proquimios, 96%), sodium chloride (Dinâmica, 99%), commercial virgin soy oil (Liza, Cargill), glutaraldehyde (Sigma Aldrich, 25%), paraformaldehyde (Sigma Aldrich, 98%), monobasic sodium phosphate (VETEC, 99%), bibasic sodium phosphate (VETEC, 99%), ethyl alcohol (VETEC, 99.5%), carbon dioxide (White Martins, 99.99%), tween 80 (Dynamic). To ensure the growth conditions of microorganisms, water, culture medium, and/or substrates, as well as glassware and laboratory utensils, were sterilized for 20 min. in an autoclave at 121 °C (Autoclave vertical Phoenix luferco).

### Fungal isolates

Four fungal isolates, *Penicillium* sp. F04, *Aspergillus* niger (F16), *Aspergillus* sp. F18, *Aspergillus* sp. F21 were kindly provided by the microbiological collection from the Microbiology sector of the Sanitation Laboratory at UFES (campus Vitória). These isolates were chosen based on previous studies [20, 21]. As presented, they show high lipase activity—5.05 ± 1.36; 2.32 ± 0.39; 0.35 ± 0.10 and 1.18 ± 0.98, respectively.

The isolates were grown on PDA plates at 25 °C, for 7 days, to assess the mycelial mass for later use, that is, in submerged fermentation for enzyme production.

The Castellani method, 1967 [22] was used to prepare the fungi isolates stocks a preservation method that consists of placing five mycelium cubes of dimensions equal to 5 × 5 mm, in a 20 mL sterile flask, which, in its turn, contains 5 mL of distilled water, also sterilized.

### Morphological characterization

The fungal isolates were first characterized by images taken directly from the medium dishes, with a 16 MP and 5MP dual camera (Samsung, SM-A305GT) and without further preparation. The microscopic characterization, on the other hand, was done by a scanning electron microscope (SEM-JEOL1600LV). For the latter, the samples were fixed for 24 h in the Karnovisk solution (Glutaraldehyde 2% + Paraformaldehyde 2.5% in Sodium 0.1 M pH 7.2). Next, for the post-fixation step, they were washed in Cacodilate buffer (0.1 mol/L; pH 7.2–7.4), in 1.25% potassium ferrocyanide solution and 1.0% buffered osmium tetroxide (Cacodilate 0.1 mol/L) at room temperature for 1 h. Once the post-fixation step was done, the crusts were washed again with 0.1 M cacodylate buffer.

Afterward, the samples were washed with cacodylate buffer solution and ultrapure water, followed by dehydration in different concentrations of ethanol. They were, then, taken to dry at a critical point (Autosandri-815, Tousimis), with subsequent gold coating in metallizer (Desk V, Denton Vaccum) for visualization in SEM. Images were taken using a 20 kV tungsten filament.

### Standardization of optimum cultivation time

The ideal cultivation time for higher enzymatic production was established through submerged cultivation in a shaker for 7 days. The growth curve, meanwhile, was outlined by removing daily aliquots of the liquid medium, while the extraction and dosage of lipases were conducted via standard curve of total proteins.

### Experimental design

For optimization of lipase production, a complete factorial planning 3^2^ was used. This design is suitable for multifactorial experiments since it works by seeking a mutual relation amongst multiple factors to identify optimum conditions for processes [23–25]. Here, the variables studied were Temperature (°C) and Inducer Concentration (%), taking into consideration its influence on enzymatic production of fungal metabolism. These variables and the respective levels approached were defined according to a bibliographic survey, as shown in Table 1.

**Table 1:** Variables and parameters studied

Variables	Levels studied	References
Temperature (°C)	25; 30; 37; 45	[26–31]
Inducer (%)	1; 1,5; 2; 4; 8	[26, 32–36]

The statistical analysis of the planning of choice was performed using the software Statistica 12.0, trial version. The matrix with the complete factorial planning (of two variables and three levels) is shown in Table 2 below:

**Table 2:** Factorial Planning Matrix 3^2^ for lipase production

Assay	Inducer (%)		Temperature (°C)
1	2 (−)		28 (−)
2	2 (−)		32 (0)
3	2 (−)		36 (+)
4	6 (+)		28 (−)
5	6 (+)		32 (0)
6	6 (+)		36 (+)
7	10 (0)		28 (−)
8	10 (0)		32 (0)
9	10 (0)		36 (+)
10	6 (+)		32 (0)
11	6 (+)		32 (0)

### Submerged phase cultivation

In order to produce lipases from each of the four fungal isolates, a circle of approximately 5 × 5 mm in diameter of the fungal colonywas inoculated with the aid of sterilized pipette tips in a 125 mL Erlenmeyer flaskcontaining 50 mL of minimum medium (MM). The MM consisted of NaCl (5.0 g L^−1^), CaCl_2_ (1.0 g L^−1^) and soybean oil (inducer) emulsified with 0.1% Tween 80 in the vortex (Phoenix luferco) and added to each one of the assays. The quantity of the inducer was defined according to the levels of the complete factorial planning. The flasks with enriched samples stirred at 110 rpm in an orbital shaker incubator (Solab, SL233) for 6 days. The levels of the inducer and temperature evaluated are shown in Table 2.

### Extraction and dosage of lipases

Following the previous step, the flasks were removed from the shaker and the sodium acetate buffer (pH 4.5) was added at a ratio of 10:1 (buffer: medium). Then they were both kept under stirring for 60 min. The resulting content was filtered through filter paper (Unifil) and centrifuged at 15,000 rpm (Sigma) for 10 min.

Ultraviolet absorption was the method employed to dose the total of proteins from the samples obtained in the previous steps. This procedure entailed pipetting the samples inside quartz cuvettes before running it through a spectrophotometer (Thermo Scientific, Genesys 10 UV Scanning) at 280 nm wavelength, according to Zaia et al. [37]. Five measurements of absorbance in different dilutions were made, all of them at wavelength equal to 280 nm to obtain the standard curve for protein dosing. The standard curve with BSA was then applied to the readings gathered from this step.

## Results and discussion

### Morphological characterization

**Fig. 1:**
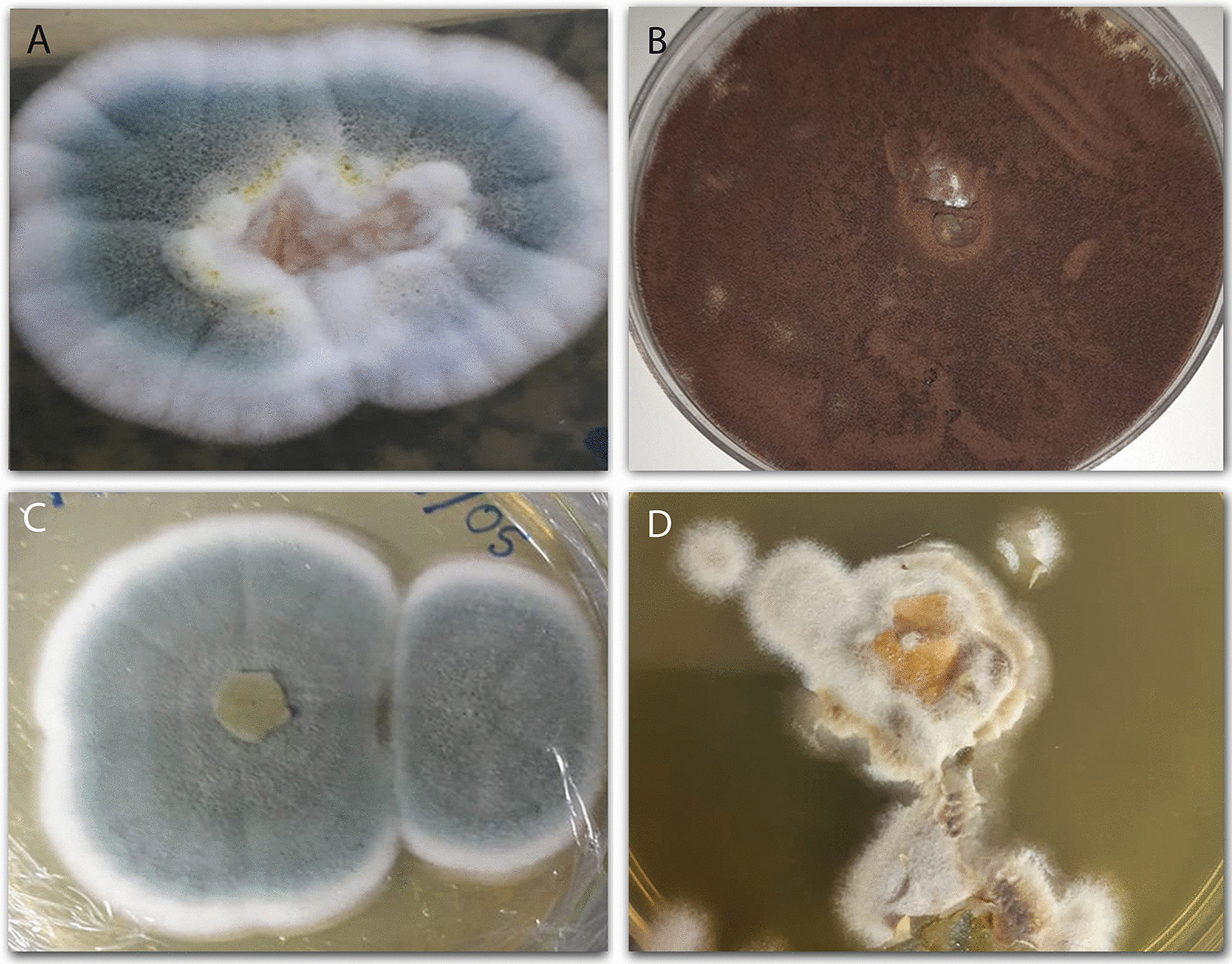
Images obtained from low magnification for observation of the coloniesof *Penicillium* sp.F04 (**A**), *Aspergillus niger* F16 (**B**), *Aspergillus* sp. F18 (**C**), and *Aspergillus* sp. F21 (**D**)

The fungal isolates used in this work were obtained from grease boxes and soils in which oily residues were found [20, 21]. The cultivation medium with the inducer (soybean oil) is selective since only organisms capable of metabolizing lipid as carbon and energy source grew on it. Growth and multiplication of microorganisms on any substrate is often considered as the first step towards its bioconversion. In Fig. 1 are the cultures of the four fungal isolates, and it is possible to observe both the appearance and the coloration of the agglomerated mycelia macroscopically. Also known as “sac fungi” due to the shape of their asci [38], the ascomycetes genera Penicillium and Aspergillus [39, 40] are represented by filamentous fungi that can be found in air, soil, vegetation, and indoor environments [41, 42].

**Fig. 2:**
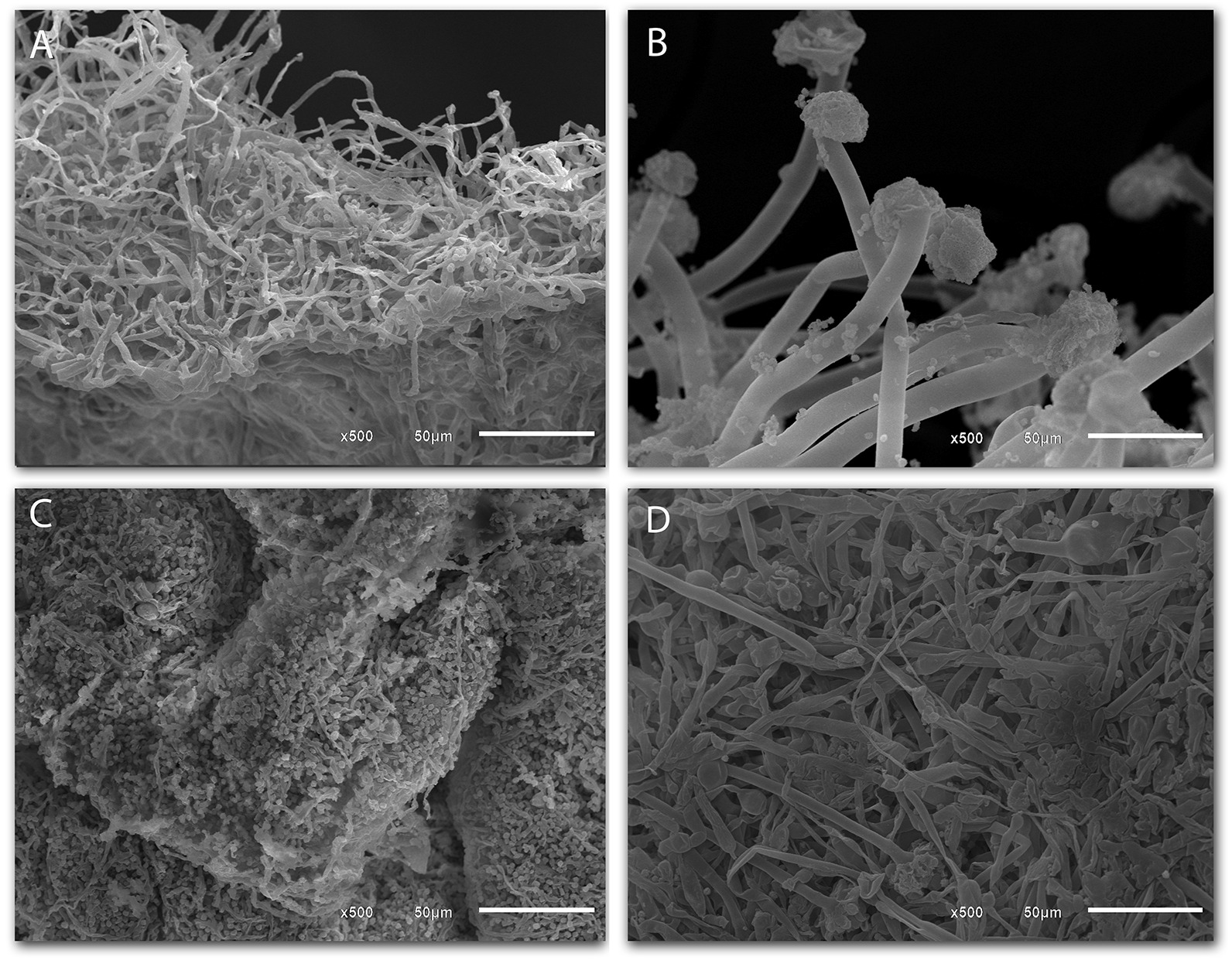
Images obtained through scanning electron microscopy (SEM) to observe the microstructures of the fungalisolates: *Penicillium* sp. (**A**), *Aspergillus* niger (**B**), *Aspergillus* sp. (**C**), and *Aspergillus* sp. (**D**)

On the other hand, the microscopic analysis of the isolates in this work was done by scanning electron microscopy, where it is possible to observe the fungal cells ultra-structures, presented in Fig. 2. Image A shown the *Penicillium* sp. F04 hyphae, whereas image B clearly shows the conidiophores and conidia of *Aspergillus niger* F16. According to Cruz [43], this last one species has globular, warty, and finely wrinkled conidia. Image C, in its turn, presents *Aspergillus* sp. F18 hyphae, and image D shows both the hyphae and conidia of the *Aspergillus* sp. F21. When making microscopic analysis of *Penicillium* species, the septate hyphae are observed. These often-anastomosed hyphae have walls devoid of pigmentation [44]. As for their conidiophores, they appear as branches of the mycelium and are mostly perpendicular to the substrate. Additionally, they are composed of a stipe that can present itself as narrow or broad [45].

### Standardization of optimal cultivation time

**Fig. 3:**
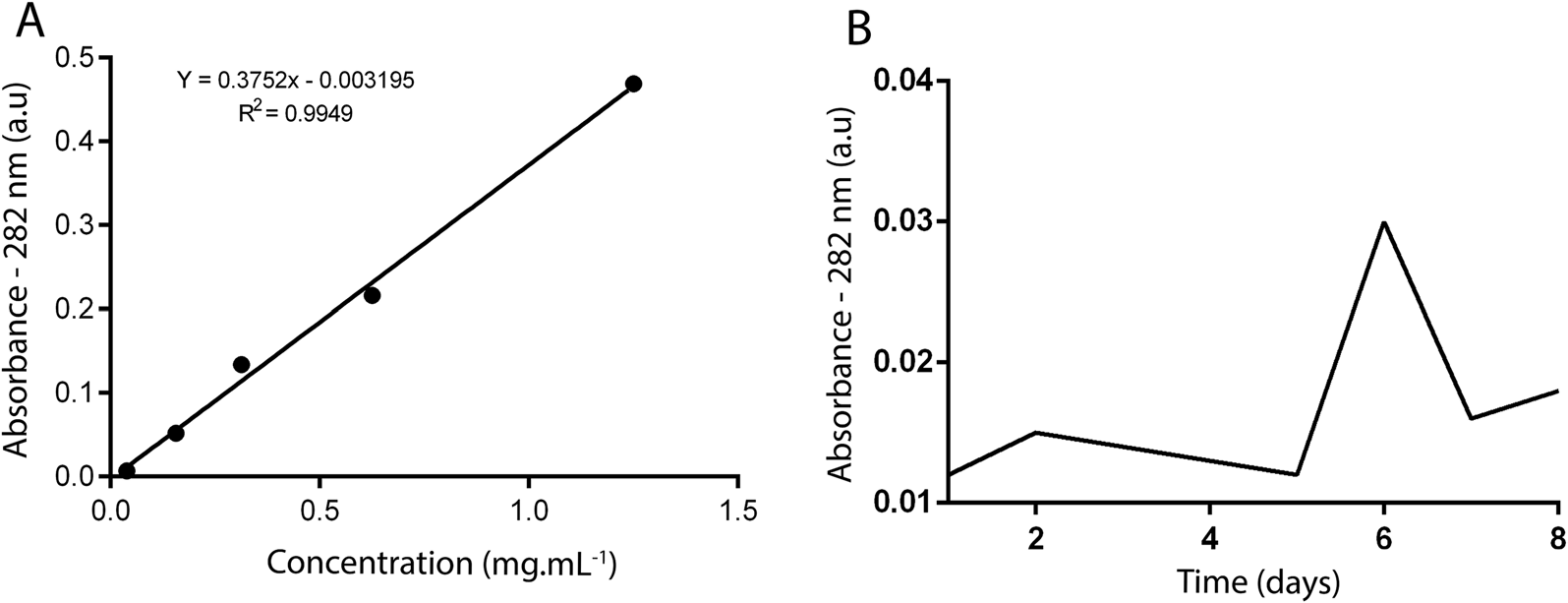
Standard curve for total protein dosing, using BSA as protein model (**A**); Optimum time curve for production of lipases by *Penicillium* sp. F04 isolate (**B**)

Determining the optimal cultivation time for enzyme production is an important study parameter because time has a direct impact on lipase production as well as process costs. For this, the growth curve based on the lipases production was determined. The BSA protein is frequently used as standard for protein quantification deriving from cellular metabolism [46]. As such, Fig. 3A shows the results of the curve and the regression equation used for the quantification of total lipases. In order to establish the optimal cultivation time, a growth curve was performed—as described in Sect. 2.2.3 of the methodology—, using the isolate *Penicillium* sp. F04 as study model. Results show that the optimal time for the enzymatic production is 6 days, with production starting on the fifth day, as indicated in Fig. 3B.

Cola et al. [47] obtained 3 to 4 days for the ideal time were using *Aspergillus* as study model to investigate the optimization of its lipase production. Kempka et al. [26], however, when optimizing the production of *Penicillium verrucosum* lipases, adopted 7 days for fermentation time. This divergence can be attributed to the varieties of each fungal isolate and conditions handled in their work, as well as the medium or inducers used on the different approaches.

### Total lipases

Factorial planning is a widely used technique when two or more independent variables (factors) are investigated. It allows a combination of all variables at all levels, thus obtaining an analysis of a single variable, subject to all combinations from the others. [48]. For assessment of the effects of each studied variables and levels—namely, temperature (28 °C, 32 °C and 36 °C) and inducer concentration (2%, 6%, and 10%)—, the response variable used was total protein dosage according to the standard curve (Fig. 3A). The results for each experiment are presented in Table 3.

**Table 3:** Total Lipases obtained for each level from the Factorial Design Matrix 3^2^

Assay	Inducer (%)	Temperature (°C)	Total lipases (µg mL^−1^)			
			F04	F16	F18	F21
1	2	28	936.7	395.2	2290.2	2425.5
2	2	32	2560.9	801.3	11,007.0	4510.0
3	2	36	2127.8	1613.4	4753.7	4320.5
4	6	28	1965.3	720.1	1396.9	1099.1
5	6	32	1586.4	1369.8	1586.4	3183.5
6	6	36	7406.6	1451.0	1911.2	2019.5
7	10	28	1830.0	1559.3	4022.7	1694.6
8	10	32	2560.9	1532.2	2371.4	1396.8
9	10	36	2209.0	1288.6	2209.0	1532.2
10	6	32	1775.9	1310.5	2127.8	3156.5
11	6	32	1770.0	1190.5	2019.5	3102.3

### Statistical analysis

The analysis of variance (ANOVA) was done with the software Statistica 12.0 trial version, with significance level at 5%, as shown in Table 4. The interactions between variables were also analyzed, being them: Linear interaction (L) or of the first order, and quadratic interaction (Q) or second order. The significant variables are shown in bold.

**Table 4:** ANOVA results for each fungal isolate analyzed: *Penicillium* sp. F04), *Aspergillus niger* F16, (*Aspergillus* sp. F18) and (*Aspergillus* sp. F21)

	SQ*	Df*	MS*	F*	p*
*Penicillium* sp. F04					
(1) Indutor (%) (L)	158,275	1	158,275	13.6338	0.066152
Indutor (%) (Q)	**6,443,756**	**1**	**6,443,756**	**555.0654**	**0.001797**
(2) Temperatura (°C) (L)	**8,193,288**	**1**	**8,193,288**	**705.7702**	**0.001414**
Temperatura (°C) (Q)	**515,061**	**1**	**515,061**	**44.3674**	**0.021805**
1L by 2L	164,877	1	164,877	14.2025	0.063751
1L by 2Q	79,138	1	79,138	6.8169	0.120703
1Q by 2L	**7,226,888**	**1**	**7,226,888**	**622.5244**	**0.001603**
1Q by 2Q	**8,930,000**	**1**	**8,930,000**	**769.2306**	**0.001297**
Error	23,218	2	11,609		
Total SS	29,321,982	10			
*Aspergillus niger* F16					
(1) Inducer (%) (L)	**410,921**	**1**	**410,921.3**	**18.48341**	**0.007721**
Inducer (%) (Q)	213	1	212.7	0.00957	0.925880
(2) Temperature (°C) (L)	**469,504**	**1**	**469,504.4**	**21.11850**	**0.005864**
Temperature (°C) (Q)	13,146	1	13,146.2	0.59132	0.476642
1L by 2L	**554,206**	**1**	**554,205.8**	**24.92840**	**0.004130**
Error	111,160	5	22,231.9		
Total SS	1,559,213	10			
*Aspergillus* sp. F18					
(1) Inducer (%) (L)	**14,876,821**	**1**	**14,876,821**	**181.2705**	**0.005471**
Inducer (%) (Q)	**17,148,068**	**1**	**17,148,068**	**208.9451**	**0.004752**
(2) Temperature (°C) (L)	225,855	1	225,855	2.7520	0.238998
Temperature (°C) (Q)	**12,774,514**	**1**	**12,774,514**	**155.6544**	**0.006363**
1L by 2L	**4,573,610**	**1**	**4,573,610**	**55.7283**	**0.017475**
1L by 2Q	**22,574,890**	**1**	**22,574,890**	**275.0696**	**0.003616**
1Q by 2L	11,957	1	11,957	0.1457	0.739419
1Q by 2Q	**6,120,944**	**1**	**6,120,944**	**74.5822**	**0.013144**
Error	164,139	2	82,070		
Total SS	76,573,189	10			
*Aspergillus* sp. F21					
(1) Inducer (%) (L)	**7,331,455**	**1**	**7,331,455**	**4287.367**	**0.000233**
Inducer (%) (Q)	**730,825**	**1**	**730,825**	**427.380**	**0.002332**
(2) Temperature (°C) (L)	**1,173,068**	**1**	**1,173,068**	**685.999**	**0.001455**
Temperature (°C) (Q)	**1,641,584**	**1**	**1,641,584**	**959.983**	**0.001040**
1L by 2L	**1,058,224**	**1**	**1,058,224**	**618.839**	**0.001612**
1L by 2Q	**610,744**	**1**	**610,744**	**357.158**	**0.002788**
1Q by 2L	976	1	976	0.571	0.528886
1Q by 2Q	**803,516**	**1**	**803,516**	**469.889**	**0.002121**
Error	3420	2	1710		
Total SS	7,331,455	1	7,331,455	4287.367	0.000233

**Fig. 4:**
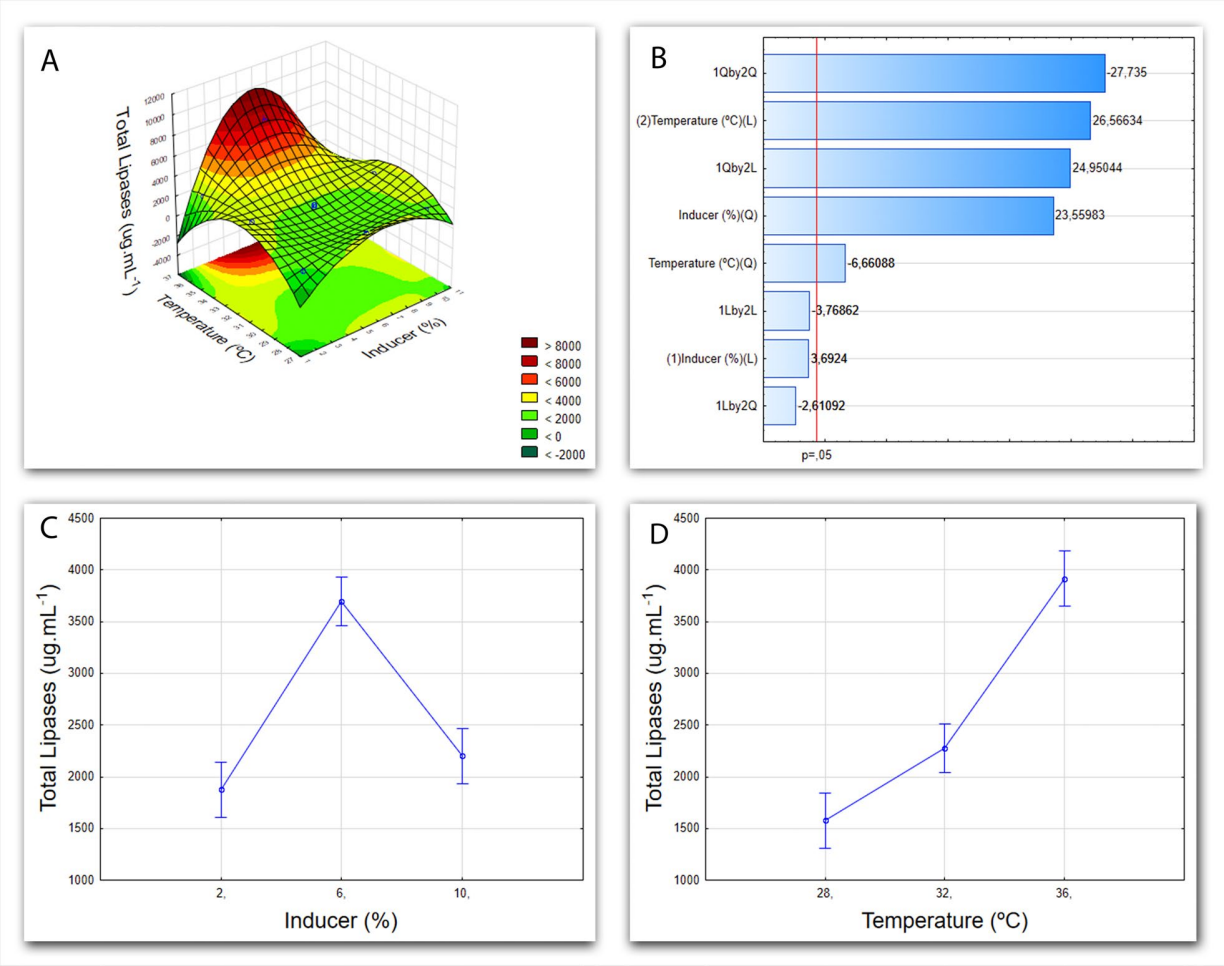
Cultivation graphs for *Penicillium* sp. F04. **A** Factorial design 3^2^ graph of the response surface, demonstrating the influence of the variables (temperature and inducer) on the response variable (total proteins). **B** Pareto Chart showing the linear (L) and quadratic (Q) effects of each of the variables tested. **C** Graph of temperature influence as a function of the response variable (total proteins). **D** Graph showing the influence of the inducer as a function of the response variable (total proteins)

**Fig. 5:**
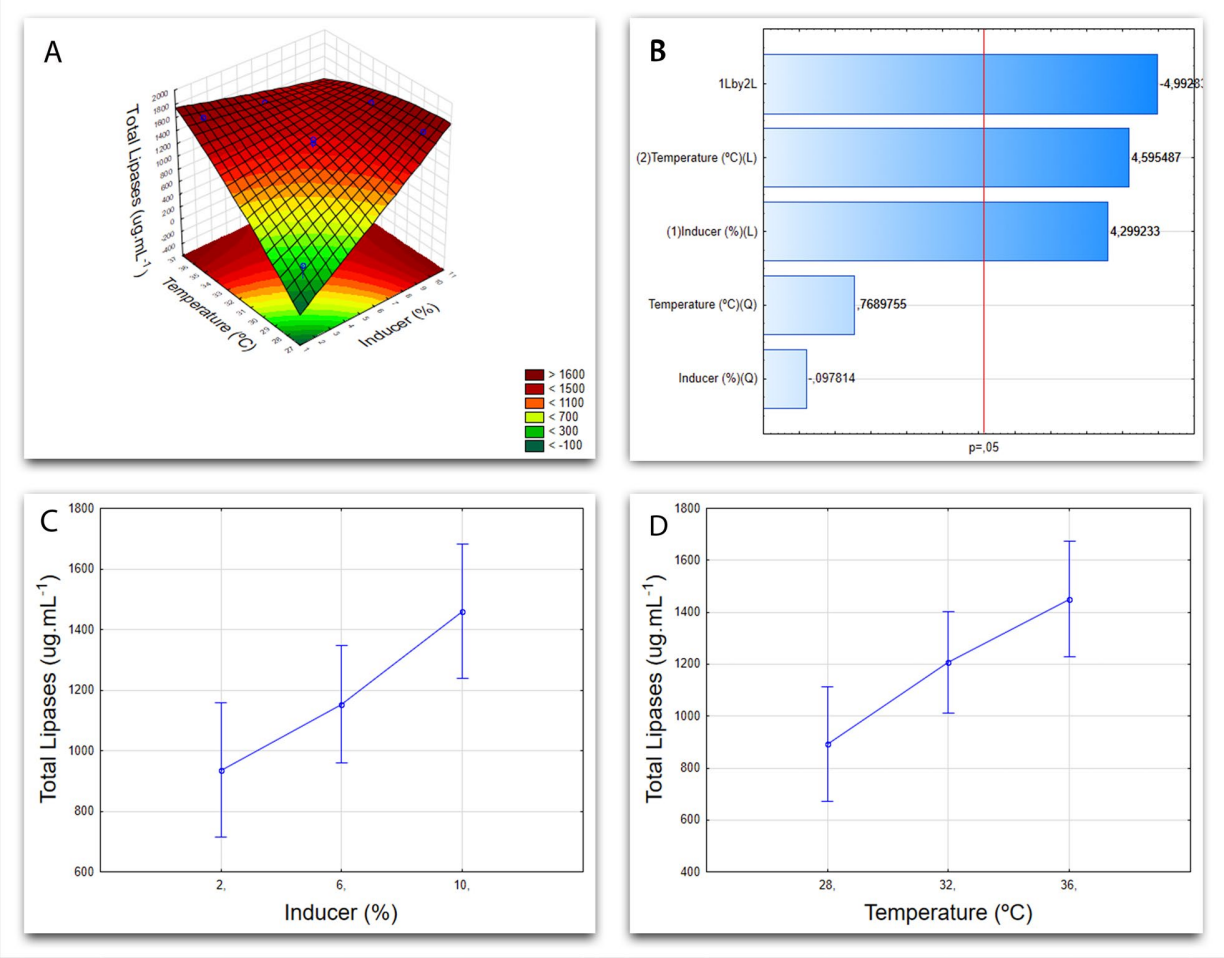
Cultivation graphs for *Aspergillus niger* F16. **A** Graph of response surface from the factorial design 3^2^, showing the influence of variables, that is, temperature and inducer, on the response variable (total proteins). **B** Pareto Chart with linear (L) and quadratic (Q) effects of each of the tested variables. **C** Graph showing the influence of temperature as a function of the response variable (total proteins). **D** Likewise, the graph shown the influence of the inducer as a function of the response variable (total proteins)

**Fig. 6:**
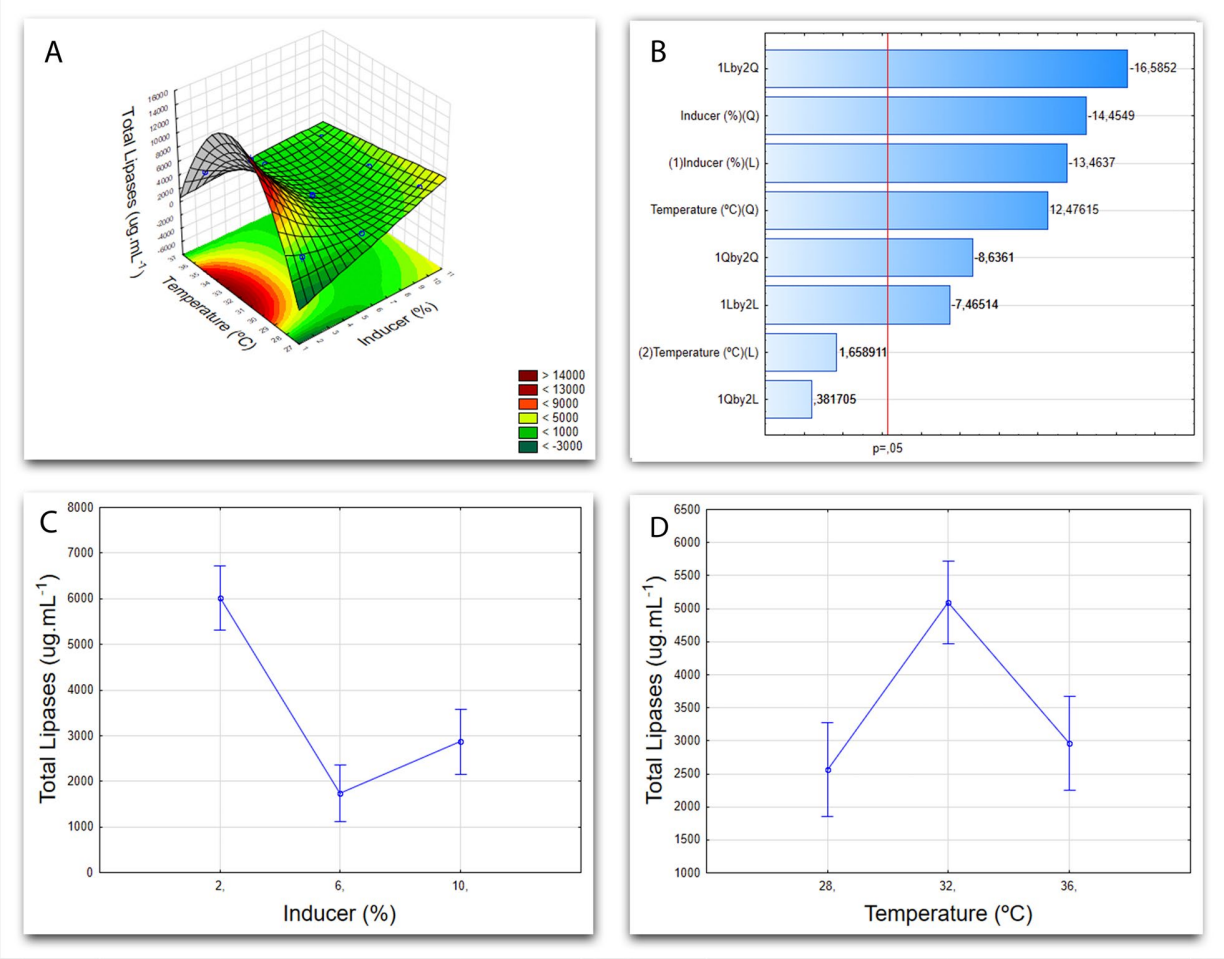
Cultivation graphs for *Aspergillus* sp. F18. **A** Factorial design 3^2^ graph of the response surface, showing the influence of variables (temperature and inducer) on the response variable (total proteins). **B** Pareto Chart showing the linear (L) and quadratic (Q) effects of each of the variables tested. **C** Graph showing the influence of temperature as a function of the response variable (total proteins). **D** Graph showing the influence of the inductor over the response variable (total proteins)

**Fig. 7:**
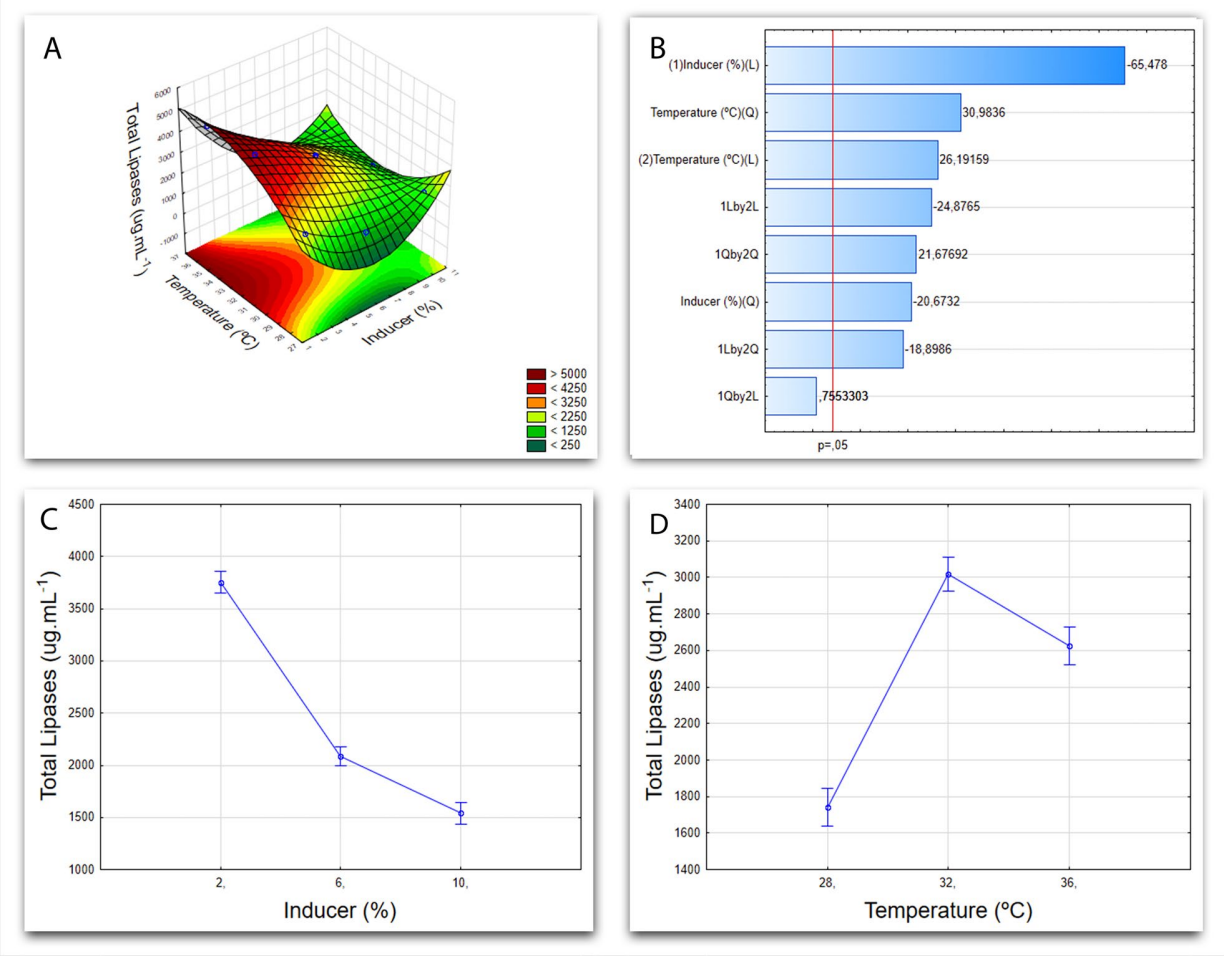
Cultivation graphics for *Aspergillus* sp. F21. **A** Response surface graph from the factorial design 32, exhibiting the variables (temperature and inducer) influence on the response variable (total proteins). **B** Pareto Chart showing the linear (L) and quadratic (Q) effects of each of the variables tested. **C** Graph showing the influence of temperature over the response variable (total proteins). **D** Graph showing the influence of the inducer in function of the response variable (total proteins)

For better visualization of both outcome and significance of the study, Figs. 4, 5, 6 and 7 follow the same logical sequence of the results attainment with the graph of the response surface (A), showing the influence of the two variables and their levels on the response variable (i.e. total lipases); the Pareto chart (B) where the effects of each variable and their first (L) and second (Q) order interactions are shown; the graphs of the individual influences of each variable, that is, temperature (C) and the inducer concentration (D).

Submerged fermentation of the *Penicillium* sp. F04 can achieve a higher yield of total lipases if the conditions of 36 °C and 6% of inducer in the medium are met. Figure 4 depicts this relation. The two variables studied were significant, and the effects are shown in the Pareto Chart (Fig. 4B). A closer look at the individual effects reveals that the best concentration for the inducer is 6% (Fig. 4C) and that the increase of lipases in the medium matches the increase in temperature (Fig. 4D).

Salwoom et al. [49], isolated, characterized, and produced lipases from *Pseudomonas* cultures from Antarctica. In their work, results concerning temperature associate higher lipase yield to cultivation at 10 °C—contrary to what is observed here. This dissonance can be explained by the difference of microorganisms employed in the respective studies. Ideal conditions, along with specificities, vary depending on the individual metabolism and its natural habitat. Due to these variations, production optimization is an element of great importance in regards to industrial-scale yield. Gutarra [50], used grains of babassu cake as a supplement to the liquid medium for cultivation and production of lipases by *Penicillium simplicissium*. However, the level of lipases was higher in the semi-synthetic medium, as the nutrients were readily available in it.

Figure 5 presents the graphs for the results obtained from the factorial design 3^2^ of the *Aspergillus niger* F16. Results reveal that not only both the highest temperature and inducer levels, 36 °C and 10%, are more significant for lipase production in this fugal isolate, which the response surface graph in image A demonstrate, but also that the temperature influence is greater than the inducer (*p* = 0.5) in submerged fermentation of *A. niger* F16, as indicated by the Pareto Chart in image B. Each variable can be separately analyzed in images C and D.

Research carried out with *Aspergillus westerdijkiae* [51] and *Aspergillus niger* [52] show that the yield of lipases produced is superior when an oily inducer is present in the medium, serving as a carbon source. Oliveira, et al. [53], when optimizing the production of *Aspergillus ibericus* lipases by fermentation in a solid-state, observed that the presence of 10.2% of lipids in the substrate was the ideal condition for achieving maximum lipase yield, similarly to the result obtained here in submerged fermentation for *Aspergillus niger* F16, which can be seen in Fig. 6.

Moreover, Colla et al. [27] studied the production of lipases by submerged fermentation and in the solid-state for both *Aspergillus flavus* and *Aspergillus niger* and found 37 °C as the ideal temperature for enzyme production in submerged fermentation, coming close to the result obtained in this work. They also described that the enzymes produced by submerged fermentation were more stable to temperature than those acquired by fermentation in a solid-state.

The best result achieved for lipase production from the *Aspergillus* sp. F18 designates as ideal conditions 32 °C for temperature and 2% of inducer concentration in the medium, which can be seen in Fig. 6A by the graph of surface response, demonstrating the interaction between variables and both of their levels. Figure 6C and D, on the other hand, exhibit each variable alone. As for Fig. 6B, the Pareto Chart is on shown, in which is possible to visualize greater influence of the inducer rather than temperature (*p* = 0.5) for submerged fermentation of *Aspergillus* sp. F18, the inducer also being more significant than the temperature to produce lipases when it comes to this isolate.

Similarly, another study conducted by Das et al. [32] using *Aspergillus* sp. sought to maximize *Aspergillus tamarii* lipase yield by optimizing the nutritional conditions of the submerged fermentation process. They found that mediums in which coconut oil (2.5%, v/v) were added achieved maximal lipase production, which is close to the results observed in the present research. Sethi et al. [28], produced *Aspergillus terreus* lipases using oily substrates in the culture medium, with the temperature at 30 °C.

And so, in Fig. 7A, the graph of surface response presents the interaction of variables, demonstrating which levels of the factors were more significant for lipase production on *Aspergillus* sp. F21 isolates: 32 °C for temperature and 2% of inducer. It is also possible to analyze each variable separately in Fig. 7C, D. Figure 7B shows the Pareto Chart for the variables, in which the greater influence of the inducer, instead of the temperature (*p* = 0.5), in the submerged fermentation of *Aspergillus* sp. F21 is evident. In this case, a lower inducer percentage leads to higher lipase yield, when temperature is average.

Now, research about the optimization of conditions of submerged fermentation for different microorganisms revealed that having 3% coconut oil in the medium, as well temperature at 30 °C, for 5 days, resulted in the highest lipase yield in *Aspergillus flavus* [54]. These very same conditions are close to the results obtained in this work, which is being shown in Fig. 7 and can be explained by both the different mediums and inducers used, in addition to the specificity of each microorganism. Rajan et al. [33], in a comparative study of submerged fermentation and solid-state fermentation for production of alkaline lipase, submitted *Aspergillus fumigatus* to submerged cultivation at 30 °C with 1% of olive oil in the medium. These values are close to the ideal levels for *Aspergillus* sp. F21 found here. Also, the yield achieved was 550,90 U.

In general, it appears that, for the variables evaluated here (temperature and concentration of the inducer), each isolate responds in a different way. However, these variables proved to be statistically significant for the experiments performed with all four fungal isolates, thus showing that the levels assessed are indeed very relevant for the standardization and optimization of fungal lipase production. The highest temperature evaluated (36 °C) was the best condition for lipase production in isolates F04 and F16, whereas the average temperature (32 °C) was the best for isolates F18 and F21. Regarding the concentration of the inducer, minimum value of 2% was ideal for isolates the F18 and F21, contrary to the others, that is, isolates F04 and F16, in which 6 and 10%, respectively, of the inducer concentration, were the optimum conditions.

## Conclusion

The results showed that the fungi obtained in this work can be a promising and sustainable alternative to produce lipases on a large scale. The factorial design using the variables temperature and concentration of the inducer enabled the optimization of lipases production. Yet, among the fungi isolates used in this study, the one which presented the greatest lipase yield was *Aspergillus* sp. F18, with 11,007 (µg mL^−1^) at 32 °C and 2% of the inducer as cultivation conditions.

## Statement of novelty

Oily residues from environmental sanitation are considered one of the main problems for wastewater treatment plants. In these residues are found several wild microorganisms adapted to lipid metabolism. In this work, we evaluate the potential of these microorganisms in the production of enzymes (lipases) that are widely demanded in the industry.

## Data Availability

The datasets used and/or analyzed during the current study are available from the corresponding author on reasonable request.
